# Phosphocholine‐containing ligands direct CRP induction of M2 macrophage polarization independent of T cell polarization: Implication for chronic inflammatory states

**DOI:** 10.1002/iid3.112

**Published:** 2016-06-20

**Authors:** JoAnn Trial, Katarzyna A. Cieslik, Mark L. Entman

**Affiliations:** ^1^ Division of Cardiovascular Sciences and the DeBakey Heart Center, Department of MedicineBaylor College of MedicineHoustonTexasUSA; ^2^ Houston Methodist HospitalHoustonTexasUSA

**Keywords:** C‐reactive protein, FcγR, macrophage

## Abstract

**Introduction:**

We studied monocyte transendothelial migration and subsequent polarization into M1/M2 macrophages in response to C‐reactive protein (CRP) with two disease‐related ligands: (1) phosphocholine (PC) and (2) multilamellar liposomes containing both unoxidized and oxidized forms of the lipid, phosphatidylcholine. These ligands differ in biological origin: PC is present on bacterial cell walls while oxidized lipids are present in atherogenic lipids.

**Methods:**

We used an in vitro model of human monocyte transendothelial migration and assessed the polarization of monocytes and T cells and signaling through Fcγ receptors in monocytes.

**Results:**

CRP without ligands did not promote M2 macrophage differentiation over background levels. However, when paired with either ligand, it increased M2 numbers. M2 differentiation was dependent on IL‐13, and in the case of CRP with PC, was associated with a Th2 response. Paradoxically, while CRP with PC initiated a Th2 response, the combination of liposomes with CRP resulted in a Th1 response without any change in Th2 numbers despite association with M2 macrophage polarization. To resolve the conundrum of an anti‐inflammatory macrophage response coexisting with a proinflammatory T cell response, we investigated signaling of CRP and its ligands through Fcγ receptors, which leads to macrophage activation independent of T cell signaling. We found that CRP plus PC acted via FcγRI, whereas CRP with liposomes bound to FcγRII. Both were activating signals as evidenced by SYK phosphorylation.

**Conclusion:**

We conclude that CRP with ligands can promote M2 macrophage differentiation to fibroblasts through FcγR activation, and this may result in an anti‐inflammatory influence despite a proinflammatory T cell environment caused by oxidized lipids. The potential relationship of this mechanism to chronic inflammatory disease is discussed.

## Introduction

Monocytes can transform into macrophages with an M2 phenotype; some of these M2 macrophages, in tissue and in in vitro models, can possess the characteristics of fibroblasts and participate in fibrosis in some organs 1, 2, 3, 4. In vivo and in vitro, serum amyloid P component (SAP) prevents the maturation of these cells and the subsequent fibrosis 2, 5. The pentraxins SAP and C‐reactive protein (CRP) function as pattern recognition molecules and opsonins through binding to Fc gamma receptors (FcγR, FCGR) (reviewed in Ref. 6). Given this commonality of function, we hypothesized that CRP and SAP would have similar anti‐fibrotic effects. We employed a human in vitro model of monocyte‐to‐M2 macrophage/fibroblast maturation that has been predictive and confirmatory of the results seen in our mouse in vivo models of tissue inflammation and fibrosis 4. In this model, human cardiac microvascular endothelial cells are used as a barrier for mononuclear leukocyte (MNL) transmigration in response to monocyte chemoattractant protein‐1 (MCP‐1, CCL2).

After transmigration, the monocytes mature sequentially into M1 proinflammatory macrophages followed by M2 anti‐inflammatory macrophages with the characteristics of fibroblasts. T cells also migrate, and sequentially differentiate into Th1 followed by a sustained Th2 phenotype. We have previously used this model to demonstrate that two forms of CRP, the native pentamer and the monomer (normally found dissociated from the pentamer by lysophospholipids on a membrane surface), have differing effects on macrophage and T cell differentiation 7. The native pentamer induced a Th2/M2 response, while the monomer induced a Th1/M1 response. Those studies were performed in the presence of fetal bovine serum in the medium to mimic a serum environment and thus potentially contained undefined lipid or other ligands for the added CRP. In the present study, we eliminated serum and sought to define more rigorously the role of the ligand in the biologic response. We used a serum‐free model with added ligands containing phosphocholine (PC, a major ligand for CRP) and multilamellar lipid vesicles containing an oxidized phosphatidylcholine.

Each of these ligands is potentially relevant to human disease. Thirty to forty percent of bacterial species in dental plaque bear PC on their cell walls 8, and periodontitis has been reported to be a risk factor for myocardial infarction 9. *Streptococcus pneumoniae*, which bears PC, causes most cases of community‐acquired pneumonia and sepsis. It also invades heart tissue, where it is associated with increased myocardial infarction and heart failure in humans and collagen deposition in animal models 10. The PC head group is also found on atherogenic‐oxidized low density lipoproteins 11, 12. CRP binds to these oxidized but not native phospholipids, which may bear some relationship to atherogenesis.

We performed these studies with native human mononuclear cells and cardiac microvascular endothelium in which the environment was rigorously controlled. We used a model that emulates in vivo models of inflammation with great fidelity and materials that are contaminant free. We found that (1) CRP alone did not affect monocyte biology but when bound to (2) PC or multilamellar lipids containing oxidized phosphatidylcholine, initiated an M2 response. Surprisingly, despite the similar response with regard to macrophage polarization, the oxidized phospholipid initiated a Th1 response in the T cell population while the PC initiated a Th2 response. Our studies suggest that the difference in the macrophage response to CRP when bound to these different ligands resulted from targeting of different Fcγ receptors and was independent of the T cell response. Thus, the response to an oxidized phospholipid, which has been associated with atherosclerosis, initiates an inflammatory Th1 response in T cells; paradoxically, it initiates an anti‐inflammatory profibrotic response in macrophage polarization. This may explain some of the controversy regarding mononuclear cell signaling by CRP and its role in atherosclerosis.

## Materials and Methods

### Reagents

RPMI1640 medium was from Invitrogen (Chicago, IL), fetal bovine serum (FBS) was from Hyclone (Pittsburgh, PA), and ITS‐X and Brefeldin A were from Sigma–Aldrich (St. Louis, MO). All cytokines, carrier‐free CRP (in Tris buffer without any other proteins for stabilization), and blocking constructs were obtained from R&D Systems (Minneapolis, MN). To block the action of IL‐13, the recombinant human IL‐13Rα2/Fc chimeric construct was used at 1 μg, as was a control recombinant human IL‐11R/Fc chimeric molecule. Phosphocholine was obtained from Sigma–Aldrich and used at 13 ng/ml. Lipids were obtained from Avanti Polar Lipids (Alabaster, AL) in chloroform, mixed and dried under argon gas in glass tubes with teflon‐lined caps, resuspended in 0.01% Tween‐20 in PBS and sonicated for 5 minutes at 80 °C in an ultrasonic heated waterbath (Fisher Scientific) to form multilamellar liposomes. We elected to use a sonicating water bath rather than a sonicator probe to avoid foaming and overheating of the sample. The unoxidized lipid was 1‐palmitoyl‐2‐oleoyl‐sn‐glycero‐3‐phosphocholine (POPC, 30 μg/mL) and the oxidized lipid was 1‐palmitoyl‐2‐azelaoyl‐sn‐glycero‐3‐phosphocholine (PAzPC). This partially oxidized lipid was chosen over the fully oxidized lysophosphatidylcholine to limit the conversion of pentameric to monomeric CRP. For fluorescence imaging, the oxidized lipid was the fluorescent 1‐palmitoyl‐2‐{6‐[(7‐nitro‐2‐1,3‐benzoxadiazol‐4‐yl)amino]hexanoyl}‐sn‐glycero‐3‐phosphocholine (NBD‐PC), used at 1% of the POPC concentration. Omeprazole was obtained from Sigma–Aldrich, and was activated at pH 5.5 for 30 min before addition at 1:1 (final 100 μM) to cells at pH 7.2. Medium brought to pH 5.5 without the addition of omeprazole was used for the vehicle control. The cells were treated for 1 h with these reagents before use in a TEM assay. Zileuton was obtained from Tocris Bioscience (Minneapolis, MN) and used at 1 μg/mL. The human Fc block, isotype controls, and blocking antibodies against FcγRs CD16/32 (FcγRII/III) and CD32 (FcγRII) were obtained from BD Pharmingen (San Diego, CA), against CD64 (FcγRI) from eBioscience (San Diego, CA), against the R131 variant of FcγRII (clone 41H16 hybridoma) from Dr. Ted Zipf (MD Anderson Cancer Center, Houston, TX, retired) and their F(ab’)_2_ fragments purified (Pierce F(ab’)_2_ Preparation kit, Thermo Scientific, Pittsburgh, PA) before addition at 10 μg per well. Intravenous gammaglobulin (Venoglobulin‐S 5% solution) was obtained from Alpha Therapeutic Corporation (Los Angeles, CA).

### In vitro transendothelial migration (TEM)

As detailed previously 2, 13, blood was obtained from healthy volunteers under a protocol approved by the Institutional Review Board of Baylor College of Medicine. ACD (Sigma) anticoagulated blood was fractionated by Ficol‐Hypaque gradient centrifugation (Histopaque‐1077; Sigma) to collect mononuclear cells (MNL). For a TEM assay, human cardiac microvascular endothelial cells (HCMEC) (Lonza (Walkersville, MD), passages 4–9) were seeded onto an insert coated with human collagen type IV (Millipore, Temecula, CA). Human MNL (in RPMI1640 with 10% FBS and antibiotic–antimycotic (Invitrogen) or without serum but with ITS‐X (Sigma–Aldrich)) were then added to each insert and the same medium including 650 ng/mL MCP‐1 for chemoattraction was added to the well below and the indicated stimuli to the insert. MNL were allowed to migrate for 16 or 96 h before fixing and counting the cells on the bottom of the well. At the end of each migration period, the migrated cells were harvested immediately for RNA or, for protein, after 6 h of exposure to Brefeldin A (Sigma).

### Immunofluorescence

Cells that transmigrated through an endothelial barrier were allowed to attach to poly D‐lysine‐coated coverslips (NeuVitro, Vancouver, WA) at the bottom of the lower well. The cells were fixed and stained with mouse anti‐CD86 (BioLegend, San Diego, CA), MRP‐14 (S100A9, BioLegend) or rabbit anti‐CD206 (Epitomics, Burlingame, CA). For staining of intracellular IL‐13, the cells were allowed to migrate onto coverslips for 7 h and then incubated for 16 h with Brefeldin A at 10 μg/mL. At the end of the incubation, the cells were stained with anti‐CD11b‐FITC (BD Pharmingen, San Jose, CA), washed and treated according to directions for the BD Fix‐Perm kit, with intracellular staining of IL‐13 (anti‐IL‐13‐PE, BD Pharmingen). After all staining procedures, the coverslips were mounted with the cell nucleus stain DAPI. Microscopy was performed on an Olympus AX70 using a QImaging Retiga 2000R camera. Colors were assigned and merged using ImageJ software (version 1.46r, NIH). For cytometry, cells were stained with antibodies against CD3 and CD4 (Becton Dickinson, Franklin Lakes, NJ), CD294 (CRTH2, BioLegend), FOXP3 (eBioscience), and IL‐1R1 and IL‐1R4 (R&D Systems). Intracellular antigens were stained by use of the Foxp3 Transcription Factor Staining Buffer Set (eBioscience, San Diego, CA). Cells were analyzed on a Cell Lab Quanta SC flow cytometer (Beckman Coulter, Miami, FL) using the Quanta Analysis software and FlowJo software (Tree Star Inc., Ashland, OR). Gating was based on comparisons of signals from the listed antibodies compared with isotype control antibodies, wherein gates were set at 1% or less of the negative control for evaluation of positive antibodies.

### Characterization of liposomes

For transmission electron microscopy, liposomes were prepared and then collected on lacey carbon copper grids (300 mesh, EMS Sciences, Hatfield, PA). After 20 min, the grids were rinsed in three changes of water. The liposomes were then fixed in 5% ionic fluid (Hitachi, Ltd, Tokyo, Japan) and rinsed five times in water. A post‐fix composed of 4% aqueous osmium tetroxide (EMS, EM grade) was applied for 10 min after which the samples were air‐dried. Samples were viewed on a Hitachi H7500 transmission electron microscope and images were captured using an AMT XR‐16 camera run by AMT Image Capture, v602.600.51 software. The accelerating voltage was 80 kV. Some samples were treated as above, but were then stained in 2% aqueous uranyl acetate. Grids alone and grids treated in the same fashion with the detergent buffer but no liposomes were used to compare for identification purposes (data not shown).

For size distribution measurements, liposomes were placed into a Zetasizer instrument (Malvern Instruments, LTS., Malvern, UK).

### Protein array

Cells were harvested and protein was isolated using Cell Lysis Buffer (RayBiotech, Norcross, GA) supplemented with Halt Protease and Phosphatase Inhibitor Cocktail (Thermo Scientific). The protein preparation (250 µg) was loaded onto human cytokine antibody array membranes (R&D Systems). Membranes were processed according to the manufacturer's instructions, images on film were scanned, and densitometry was measured by ImageJ software. Data are expressed as integrated pixel density normalized to the positive plate controls and with the background subtracted.

### ELISA

Culture supernatants from cells 4 days post‐TEM were collected and concentrated sevenfold using 5000 MW cutoff columns (Sartorius, Goettingen, Germany). The supernatants were assayed for IL‐10 and TGF‐β using high sensitivity ELISA kits from RayBiotech.

### RNA isolation and transcriptional expression

For each donor set, there was a control group made up of MNLs that were cultured with the activators phorbol myristate acetate and calcium ionomycin for 3 days but had no contact with the TEM model. These samples expressed all gene products listed, so that the experimental samples could be compared to a donor‐specific positive control (% of control as listed in the figures).

Total RNA was isolated with TRIzol reagent (Life Technologies). Complimentary DNA was synthesized from 1 µg of RNA with a Verso cDNA synthesis kit (Thermo Scientific) using random hexamer and oligo‐dT primers (3:1). Real‐time PCR amplification reactions were performed with SsoAdvanced SYBR Green Supermix (BioRad, Hercules, CA) in triplicate using a CFX96 thermal cycler (BioRad). Cycling conditions (annealing temperature) were optimized for each primer pair. Gene expression was measured by the ΔΔCT method and was normalized to HPRT RNA levels. Primers were designed and evaluated according to MIQE guidelines 14. Primer sequences are (all in 5′‐3′):

ARG1: ACCTGCCCTTTGCTGACATCCC and ACTTCTGCCACCTTGCCAGC

FOXP3: ACAGAAGCAGCGTCAGTACC and CGGGGTATTTTTGGCAAGGC

GATA3: CCCCAAGAACAGCTCGTTTA and CTGCAAAAATGCAAGTCGAA

HPRT‐1: GACCAGTCAACAGGGGACAT and CTTGCGACCTTGACCATCTT

IL‐13: CAATGGCAGCATGGTATGG and AGAATCCGCTCAGCATCC

NOS2: CAGCATGTACCCTCGGTTCT and GGGGATCTGAATGTGCTGTT

TBET: CCCTATCCTTCCAGTGGTGA and TCAGTCCTTCATCCGTTTCC

### In‐cell Western assay

MNLs were placed into a 96‐well plate at 3 × 10^5^ in serum‐free RPMI1640 and allowed to adhere for 1 h before stimulation with CRP (5 μg), PC (12 ng), or liposomes (30 μg) singly or in combination. Two minutes after stimulation, the cells were fixed and processed with the In‐Cell Western Assay Kits I and II (LI‐COR, Lincoln, NE) using primary antibodies against phospho‐SYK (Tyr323) and total SYK (Cell Signaling, Boston, MA) at 1:200 and the IRDye 800CW‐labeled secondary antibody (from the kit) at 1:500. Plates were read on an Odyssey Infrared Imaging System (LI‐COR) using the Sapphire700 dye for cell number normalization.

### Statistical analysis

All data are expressed as mean ± SE. Since data were not always normally distributed, the nonparametric Mann–Whitney U‐test was used for comparison of two data sets, whereas the Tukey–Kramer Multiple Comparisons Test was used for more than two. A *p*‐value <0.05 was considered statistically significant.

## Results

### M2 macrophage differentiation after TEM with phosphocholine and CRP

**Figure 1:**
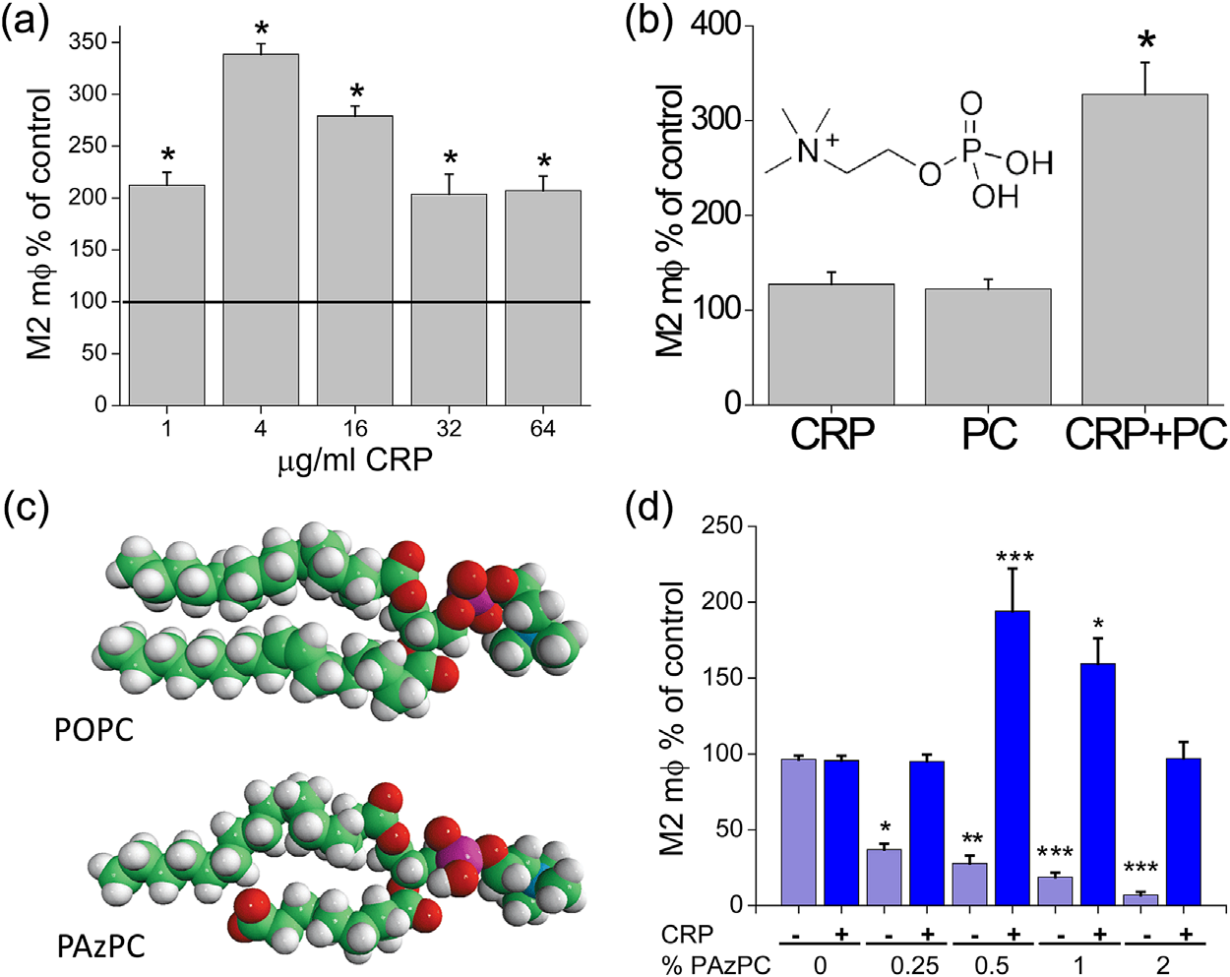
Counts of monocytes maturing into M2 macrophages after TEM through HCMEC. (a) MNLs in RPMI1640 with 10% FBS were untreated or treated with the indicated amount of CRP. (b) MNLs in RPMI1640 with ITS‐X and no serum were treated with 5 μg/mL CRP or 12 ng/mL phosphocholine (PC) or both. (c) Structures of POPC, an unoxidized lipid, and PAzPC, an oxidized lipid. (d) Counts of M2 macrophages post‐TEM with vehicle or liposomes formed with POPC and the indicated % content of PAzPC with and without CRP. Data were normalized to numbers from untreated wells. (*n* = 6 in triplicate, *p < 0.05, **p < 0.01, ***p < 0.001).

In a TEM assay that used medium with serum, a wide range of CRP concentrations promoted M2 differentiation, with no significant differences between doses, indicating the possibility of saturating the system at low doses but without toxicity or other adverse consequences at higher doses (Fig. 1a). Since serum may contain CRP ligands, we tested the hypothesis that ligand binding permits or enhances the M2‐promoting potential of CRP. We performed the TEM assay using serum‐free medium and 5 μg/mL of CRP (in the low range used in the experiments in Fig. 1a), and added the most chemically simple ligand known for CRP, phosphocholine (PC, phosphorylcholine, structure shown in Fig. 1b inset) 15. At equimolar concentrations, each component alone showed no significant activity, but the combination demonstrated increased activity (Fig. 1b).

### M2 macrophage differentiation after TEM with liposomes and CRP

Because CRP can bind to the PC moiety on more complex molecules such as lipids, we tested multilamellar liposomes, with and without CRP, in the TEM assay. To model the effect of lipid oxidation in a defined way, we incorporated small amounts of an oxidized phospholipid with a PC head group (PAzPC) into the liposomes with an unoxidized lipid (phosphatidylcholine, POPC; structures shown in Fig. 1c) to make a reproducible defined reagent. The liposomes incorporating the oxidized lipid at various concentrations suppressed monocyte fibroblast differentiation, and that was opposed by the addition of CRP for all concentrations (Fig. 1d). The combination of CRP with the oxidized lipid at some concentrations also increased the number of monocytic fibroblasts over background, as was observed for the combination of CRP and PC (Fig. 1b). All further experiments were done with liposomes formed of POPC and 1% PAzPC. Their mean size was 139 ± 1.7 nm and their index of polydispersity was 0.3 ± 0.01 (see Supplementary Fig. S1a), a size range optimal for phagocytosis (reviewed in Refs. 16, 17). Transmission electron microscopy showed them to be round to ovoid structures with typical lipid osmium staining and uranyl acetate shadowing in the size range 50–200 nm (Supplementary Fig. S1b and c). Liposomes, when made with a fluorescent‐oxidized lipid (NBD‐PC), could be seen internalized by macrophages (Supplementary Fig. S1d).

### Liposomes containing oxidized lipids promote M2 macrophage differentiation in the presence of CRP

**Figure 2:**
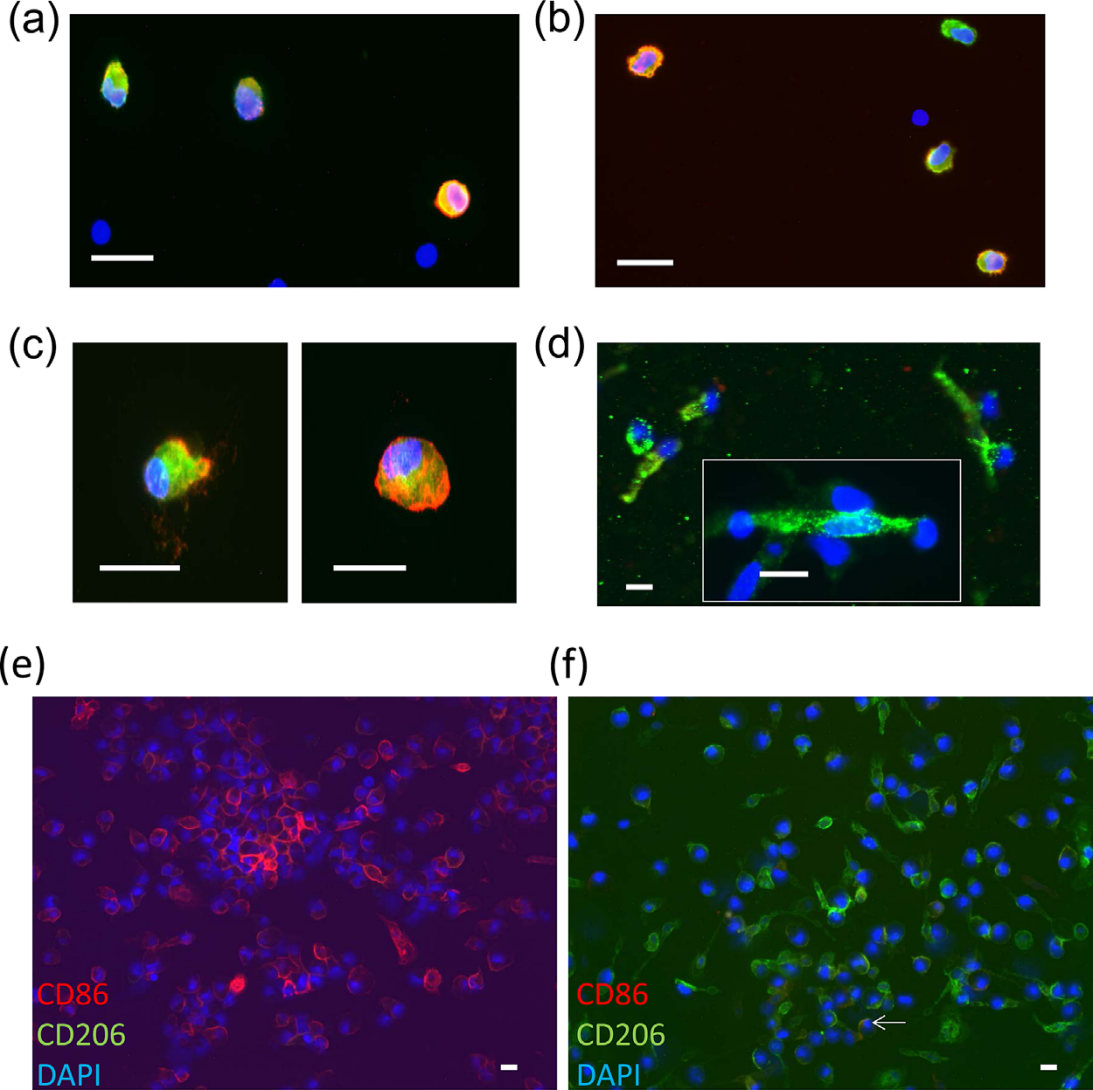
Immunofluorescence of macrophages post‐TEM after treatment with liposomes with and without CRP. (a) Cells treated with liposomes, 16 h of TEM, labeled with CD86 (red), MRP14 (green), and DAPI (blue). (b) Cells treated with liposomes and CRP, 16 h of TEM, labeled with CD86, MRP14, and DAPI. (c) Cells treated with liposomes, migrated 96 h of TEM, labeled with CD86, MRP14, and DAPI. (d) Cells treated with liposomes and CRP, migrated 96 h of TEM, labeled with CD86, CD206 (green), and DAPI. (e) Cells treated with liposomes only, migrated for 96 h of TEM, labeled with CD86 (red) and CD206 (green) at lower magnification than Fig. 2a–d. (f) Cells treated with liposomes and CRP, migrated for 96 h of TEM, and labeled as in (e), with arrow identifying a CD86^+^ cell. (scale bar = 10 μm for (a–d) and 20 μm for (e and f)).

To characterize the differentiation of the macrophages treated with liposomes with and without CRP, we stained them by immunofluorescence for markers of M1 or M2 macrophages 18. Within 16 h after the initiation of transmigration, all of the transmigrated macrophages under both conditions were in an M1 state as indicated by staining with antibodies against the markers CD86 and MRP14 (S100A9) (Fig. 2a and b). At the 4‐day harvest time, the liposome only treated transmigrated macrophages were still in the M1 state (Fig. 2c and e), but most macrophages treated with both liposomes and CRP were in an M2 phenotype, with a CD86^neg^CD206^+^ phenotype and a spindle shape with an oval nucleus (Fig. 2d and f). We have previously reported that these cells are positive for the fibroblast markers collagen type I, prolylhydroxylase, and discoidin domain receptor 2, a receptor for collagen 3, 4, 13. Counts for liposome only cultures at 96 h of TEM yielded no CD206^+^ cells and 92 ± 3% CD86^+^ cells of total cells (out of 889 counted from three donors). Counts for liposome plus CRP cultures yielded 72 ± 5% CD206^+^, 3 ± 1% CD86^+^, and 8 ± 1% double positive cells out of 667 total cells counted.

**Figure 3:**
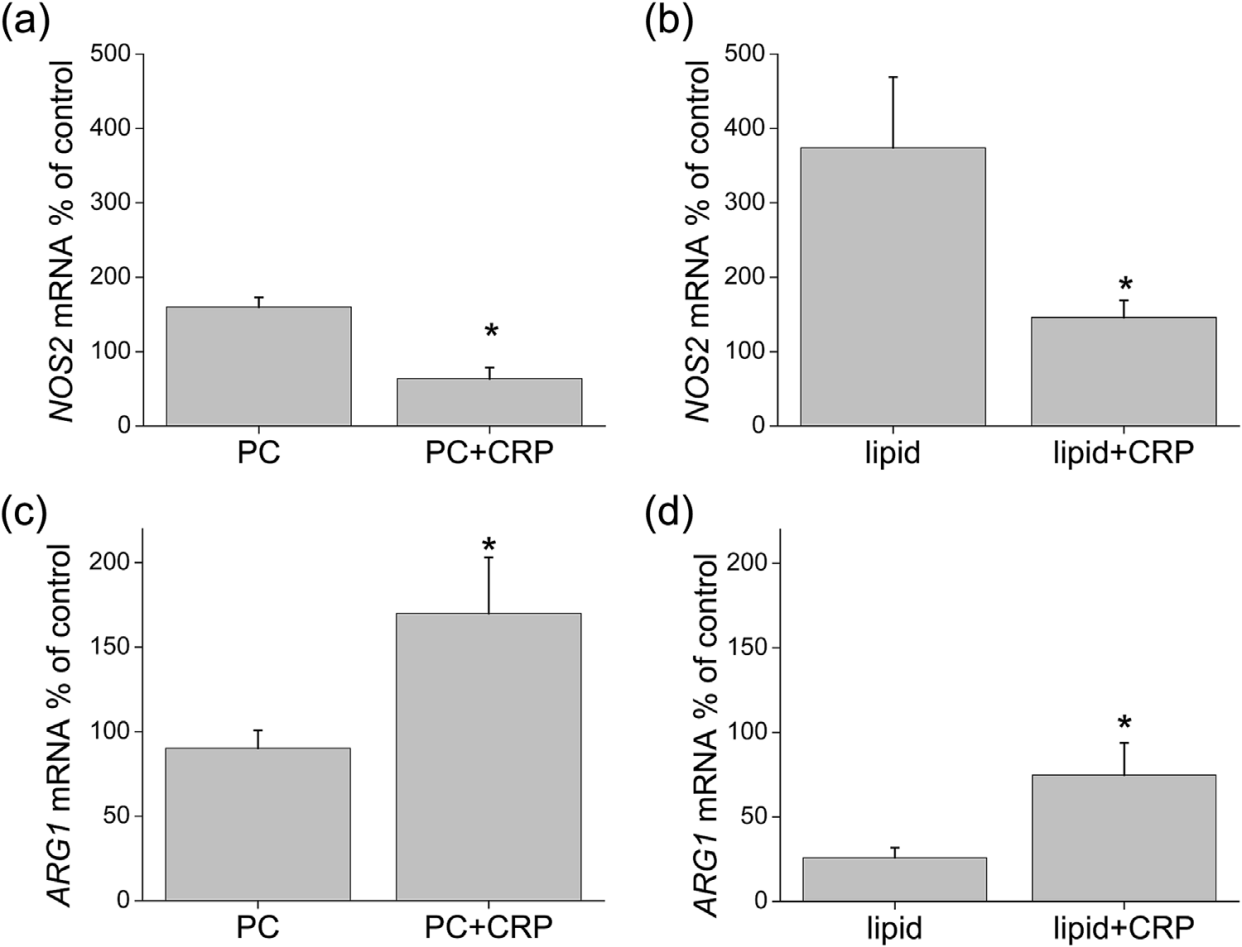
Levels of mRNA measured by qPCR expressed as a percent of expression by MNLs cultured with the mitogen/activation stimuli phorbol myristate acetate and calcium ionomycin. Levels of *NOS2* mRNA in cells treated during TEM with (a) PC or PC + CRP (*n* = 5) or (b) liposomes (lipid) ± CRP (*n* = 5). Levels of *arginase 1* (*ARG1*) mRNA in cells treated with (c) PC or PC + CRP (*n* = 5) or (d) liposomes (lipid) ± CRP (*n* = 6, *p < 0.05).

We further defined the status of the macrophages by testing for the gene expression levels of factors associated with M1 versus M2 differentiation. The data are expressed as the percent of gene expression in a control population of cells from the same donor activated with phorbol myristate acetate and calcium ionomycin (designated as 100%). We employed this method to ensure measurable levels of mRNA for each gene in our control preparations. We measured the mRNA levels of *NOS2* (*INOS*), an M1 marker 19, 20, in 4‐day migrated cells treated with the indicated reagents, and found that CRP reduced the levels seen in cells treated with PC or liposomes (lipid, Fig. 3a and b). The levels of *arginase 1* (*ARG1*, an M2 marker 21, 22, 23), by contrast, were increased by CRP (Fig. 3c and d), confirming the results from microscopy (Fig. 2).

**Figure 4:**
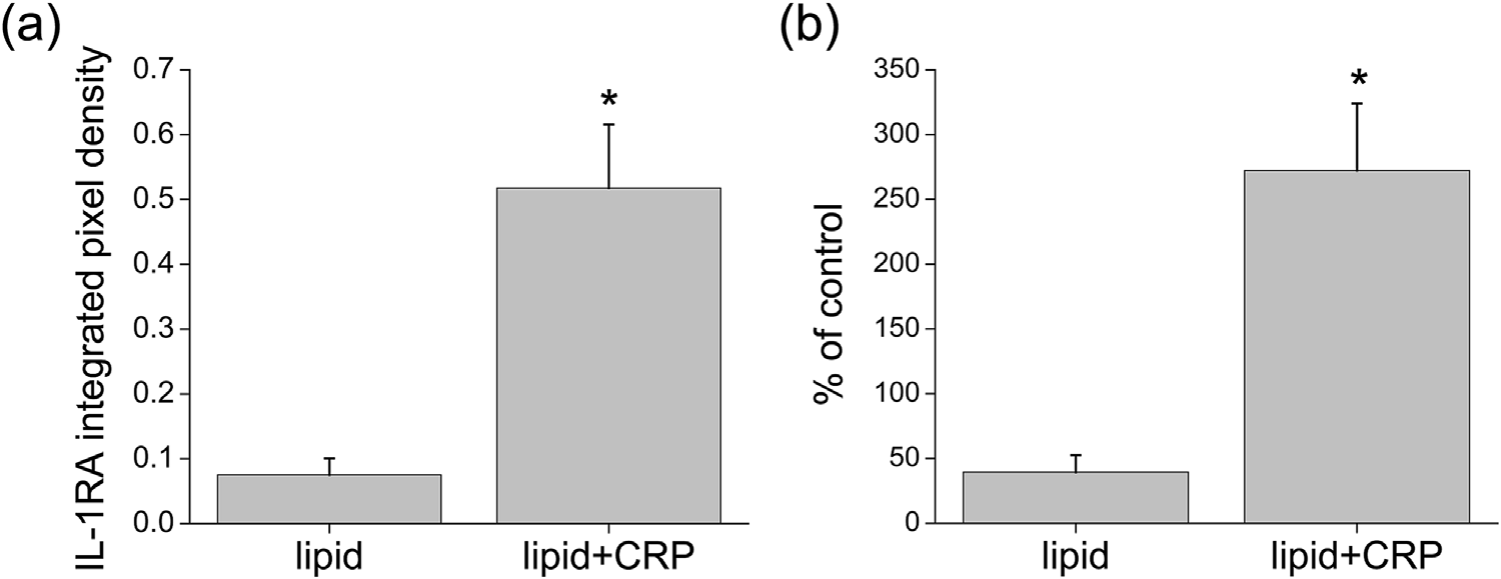
Levels of IL‐1RA in cells post TEM treated with liposomes (lipid) ± CRP measured by protein array. Integrated pixel density of the spots is shown in (a) and the data expressed as a percent of an untreated control is shown in (b). Cells were treated with Brefeldin A for 6 h before lysis to prevent secretion of the protein (*n* = 4, *p < 0.01).

Because M2 macrophages are associated with an anti‐inflammatory influence only under some conditions 18, we measured the levels of an anti‐inflammatory agent made by macrophages, IL‐1 receptor antagonist (IL‐1RA) 24 in the response to liposomes. The levels in the liposome‐treated cultures were low (Fig. 4a), even lower than those for untreated samples (Fig. 4b), perhaps because of the proinflammatory effect of this treatment. The addition of CRP significantly increased those levels, even above the control (Fig. 4a and b).

### IL‐13 signaling is critical for CRP‐induced M2 maturation

**Figure 5:**
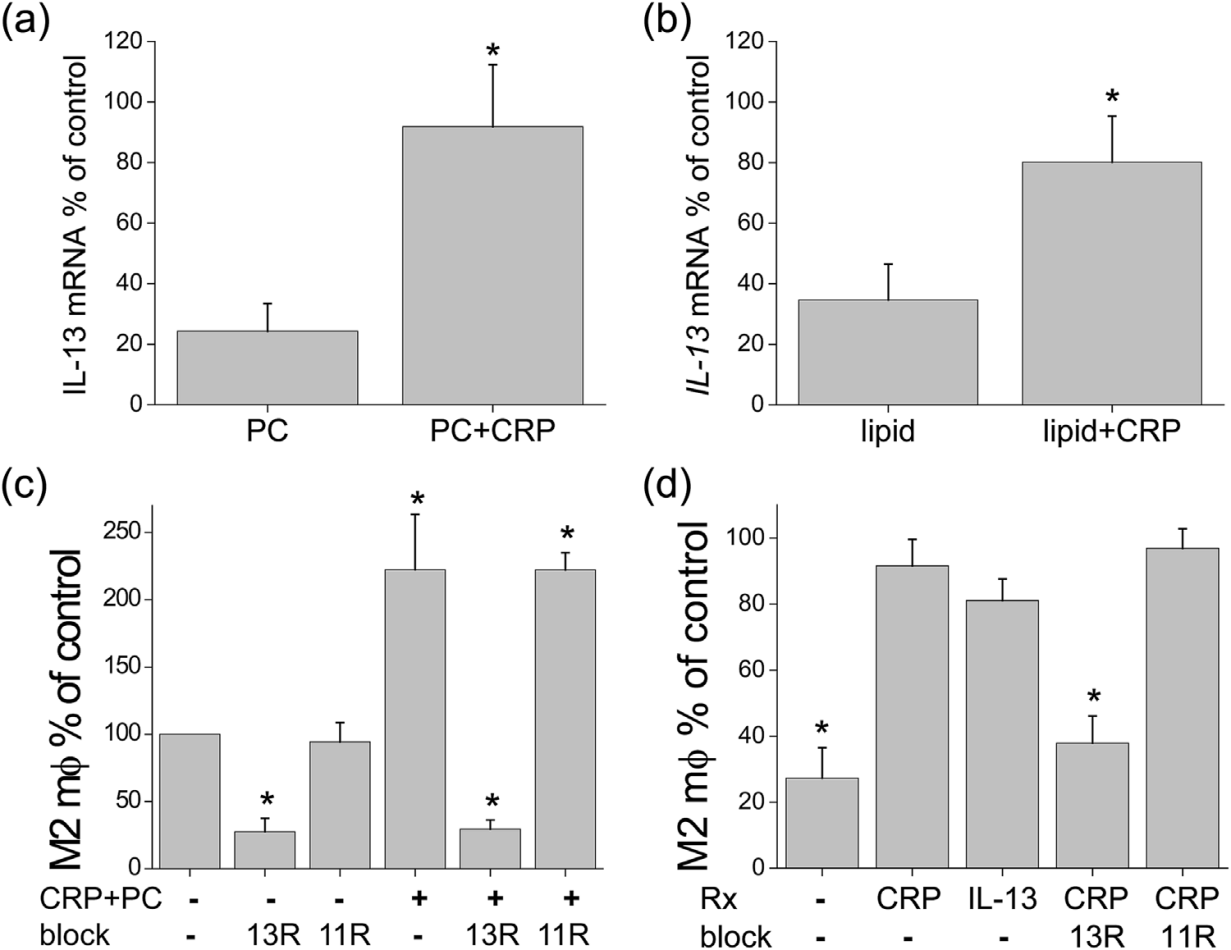
Role of IL‐13 in the observed effects on M2 differentiation. Levels of *IL‐13* mRNA measured by qPCR expressed as a percent of expression by MNLs cultured with the mitogen/activation stimuli phorbol myristate acetate and calcium ionomycin in cells treated with (a) PC ± CRP (*n* = 5) or (b) liposomes (lipid) ± CRP (*n* = 4). Counts of M2 macrophages after TEM with (c) PC or (d) liposomes and further treatments with CRP, IL‐13 (10 ng) and the blocking agent IL‐13Rα2/Fc construct (13R) or its control IL‐11R/Fc construct (11R) (*n* = 4 in triplicate, *p < 0.05).

We reported that IL‐13 promotes the differentiation of monocytes into M2 fibroblasts after TEM 4, so we investigated the presence of this cytokine in the CRP model. We measured mRNA levels of IL‐13 in cells under various treatments, and found that there was an increase with CRP whether the treatment was with PC (Fig. 5a) or liposomes (Fig. 5b). The data were expressed as a percent of the amount found in mitogen‐activated controls, a theoretical maximum positive control. To prove the role of IL‐13 in the monocyte differentiation, we used blocking agents against IL‐13 in the TEM assay. We used a chimeric molecule composed of the IL‐13 receptor α2 (IL‐13Rα2) plus the Fc portion of IgG (shown as 13R) to sequester secreted IL‐13 in the model. A similarly engineered chimera of the IL‐11 receptor was used as a control (shown as 11R). Interestingly, the IL‐13 block decreased even the background levels of M2 macrophages (Fig. 5c), as well as decreasing the enhanced numbers induced by CRP. With liposome treatment, the expected decrease in M2 numbers was seen, and this was countered by both CRP and recombinant IL‐13 (Fig. 5d). The CRP effect was blocked by 13R down to the level of liposome treatment only. We also used several drugs known to block the effects of IL‐13, the proton pump inhibitor omeprazole 25 and the leukotriene inhibitor zileuton 26, 27. Both inhibited, to different degrees, the numbers of M2 macrophages seen with CRP plus FBS (see Supplementary Figs. S2a and b). The combination of omeprazole and zileuton produced no additive effect (see Supplementary Fig. S2c). We next investigated possible origins of the generated IL‐13. Immunofluorescence of migrated adherent cells migrated for 24 h revealed CD11b^+^ cells accumulating cytoplasmic IL‐13 protein after 16 h of Brefeldin A treatment (see Supplementary Fig. S3).

### T cell responses are not required for the observed M2 maturation

**Figure 6:**
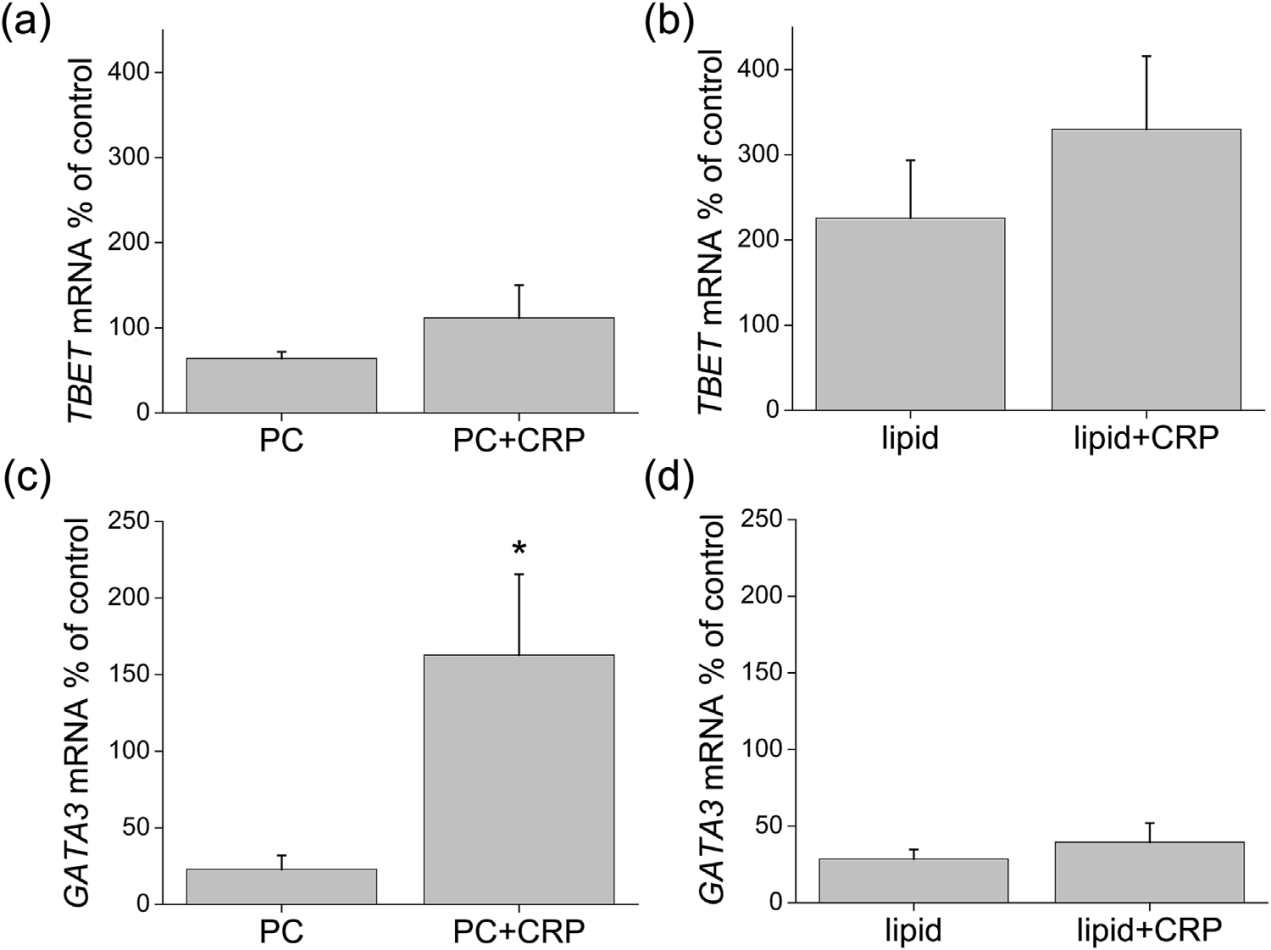
T cell subset mRNA measured by qPCR in cells post‐TEM as a percent of expression by MNLs cultured with the mitogen/activation stimuli phorbol myristate acetate and calcium ionomycin. *TBET* mRNA was measured in cells treated with (a) PC or PC + CRP (*n* = 5) or (b) liposomes (lipid) ± CRP (*n* = 6). *GATA3* mRNA was measured in cells treated with (c) PC or PC + CRP (*n* = 5) or (d) liposomes (lipid) ± CRP (*n* = 6). (*p < 0.05).

**Figure 7:**
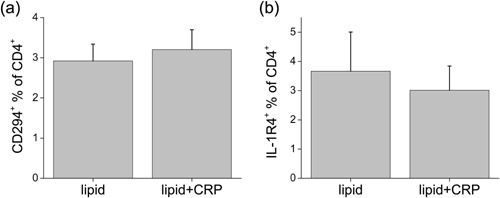
Measurement of Th2 cells by flow cytometry. After 4 days of TEM, cells were harvested and stained for immunofluorescence using antibodies against (a) CD294 and (b) IL‐1R4 measured as percentages of the total CD3^+^CD4^+^ T cells treated with liposomes (lipid) ± CRP (*n* = 5).

IL‐13 can also derive from a type 2 lymphocyte immune response 28, 29, 30, and therefore we investigated T cell subsets present in this model. We assessed gene expression of *TBET* for Th1 cells 31 and *GATA3* for Th2 and other type 2 cells 32, 33, 34, 35. We found no differences in gene expression of *TBET* with CRP treatment, although the levels were much higher than controls in cells treated with liposomes than with PC (Fig. 6a and b). With respect to *GATA3*, a dramatic increase with CRP added to PC (Fig. 6c) was not matched by any increase with CRP added to liposomes (Fig. 6d). Further confirmation of the lack of an increased type 2 response when liposomes were present was obtained by flow cytometry of cells harvested from the TEM assay. We used antibodies against CD294 (CRTH2, Fig. 7a) and IL‐1 receptor 4 (IL‐1R4, Fig. 7b) to assess Th2 cells 36, 37 and found low numbers in liposome‐treated cultures (equivalent to those found in peripheral blood), and these were not increased by the addition of CRP. Representative histograms of these and other T cell markers are shown in Supplementary Figure S5a–d.

Another way in which an anti‐inflammatory state can be promoted is via regulatory T cells (Treg) cells, and so mRNA levels were measured for the Treg transcription factor *FOXP3* 38, which did not change with the addition of CRP to liposomes (see Supplementary Fig. S4a). We also measured the number of CD4^+^FOXP3^+^ cells by cytometry; this did not change with CRP (see Supplementary Fig. S4b), and none of the FOXP3^+^ cells were positive for TGF‐β (data not shown). We measured the numbers of cells positive for IL‐1 receptor 1 (IL‐1R1) 39 and again saw no difference with CRP (see Supplementary Fig. S4c). Finally, we measured by ELISA several Treg‐associated anti‐inflammatory cytokines released into the culture supernatant. No positive signal was obtained for TGF‐β. IL‐10 was found in the supernatants, but not differently with CRP (see Supplementary Fig. S4d).

### Signaling via FcγR is critical for the observed M2 differentiation but varies with CRP ligand

**Figure 8:**
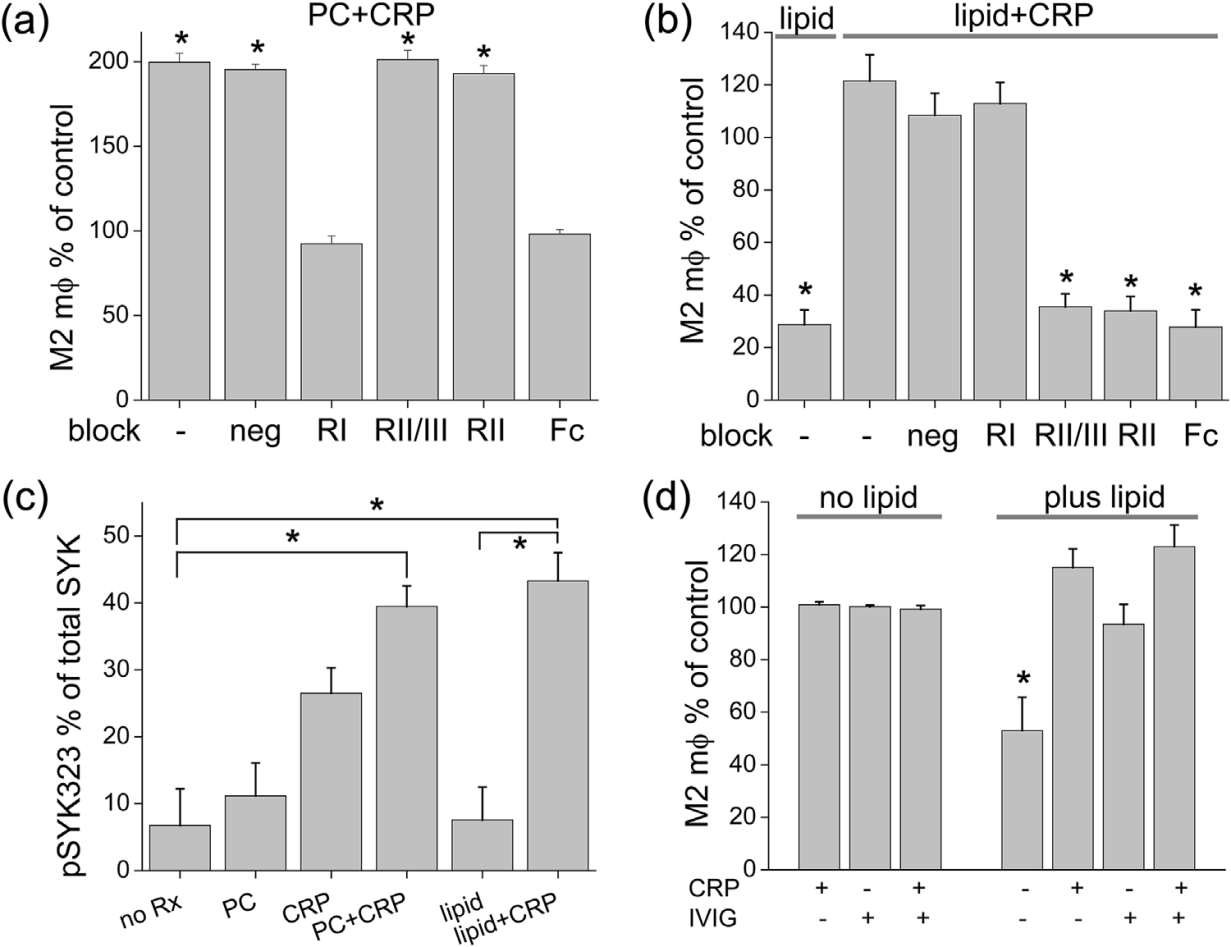
Fc receptor engagement and signaling by CRP and its ligands. Fc receptors were blocked by 10 μg/mL F(ab’)_2_ preparations of antibodies against specific receptors or combinations of receptors. RI (CD64), RII (CD32, clone 41H16), and RII/III (CD16/32) represent blocks against FcγRI‐III, and human Fc block acts against all receptors as a positive control. Counts of M2 macrophages were performed after 4 days of TEM after treatment with (a) PC + CRP (*n* = 4 in triplicate, *p < 0.05) or (b) liposomes (lipid) alone or with CRP (*n* = 5 in triplicate, *p < 0.01). (c) To measure activation of cells via FcR, the phosphorylation of SYK was measured by an In‐cell Western assay, here expressed as the percentage of the total SYK that was phosphorylated at position 323 (pSYK323) (*n* = 5, *p < 0.05). (d) Cells were treated with or without liposomes in the presence of CRP or 10 μg of IVIG or both (*n* = 5, *p < 0.001).

Since T cell responses could not explain the effects of CRP on the liposome response, and indeed should have opposed it, we sought a mechanism for M2 differentiation that could be T cell independent. Since CRP has been reported to bind to IgG Fc receptors 40, we used antibodies against FcγR to block the observed effects of CRP. All of the blocking antibodies were made into F(ab’)_2_ fragments to keep their antigen‐binding sites intact but prevent any interaction with FcγR through their Fc portions. We used human Fc block as a positive control to stop interactions with all FcγR, and antibodies specific for one or more FcγR to block individual types. This included clone 41H16, an antibody directed against the R131 variant in FcγRII 41 that binds CRP preferentially compared with H131 (which binds IgG2 preferentially) 42. When these antibodies were used, the response to PC plus CRP was blocked only by the antibody against FcγRI (CD64, Fig. 8a). However, this antibody had no effect on the response against liposomes plus CRP, in which the blocking agents targeting FcγRII (CD32) were effective (Fig. 8b). To test if the interactions of the CRP‐ligand combinations resulted in cell signaling, we measured the level of phosphorylation of SYK, a kinase downstream of activating FcγRs 43. The complexes of CRP with either PC or liposomes were particularly effective in accomplishing SYK phosphorylation (Fig. 8c). As with CRP, the addition of intravenous gamma globulin (IVIG) also opposed the suppression of M2 differentiation by liposomes (Fig. 8d).

### Endothelial cell signaling is not critical for M2 maturation

Since CRP is reported to interact with endothelial cells, we asked if those cells present in this system could have contributed to the observed results via FcγRII activation. We measured binding of the 41H16 antibody to the endothelial cells used in the cultures and the monocytes in our MNL preparations. No binding of the antibody was seen to the endothelial cells by flow cytometry (see Supplementary Fig. S6a), whereas the monocytes were positive (see Supplementary Fig. S6b). All donors used for these experiments were positive for 41H16 binding (data not shown).

## Discussion

We have previously reported that our human in vitro model of M2 macrophage differentiation gives comparable findings to our mouse in vivo models of cardiac inflammation and fibrosis mediated by monocyte‐derived M2 macrophage/fibroblasts 1, 2, 3. Our finding here that CRP increased M2 differentiation into fibroblasts contrasts with our previous studies that SAP, a similar pentraxin, had the opposite effect 2. Indeed, in humans, SAP is anti‐fibrotic 44, 45, which is consistent with our results in the mouse and in our human in vitro model. Although both pentraxins bind to FcγRs, these disparate effects have been reported to be due to the ability of SAP to inhibit fibrosis via dendritic cell‐specific intercellular adhesion molecule‐3‐grabbing non‐integrin (DC‐SIGN/CD209), to which CRP does not bind 46. Other reports about the effects of CRP on fibrosis, inflammation, atherogenesis, cardiovascular disease, and monocyte/macrophage responses are conflicting (possible reasons for which are reviewed in Scirica and Morrow 47). Some of these reasons are the manner of CRP preparation, level of administration, contaminants, and animal models. We elected to use a simplified system that bypassed these issues by using a recombinant CRP preparation from mammalian cells to avoid (1) bacterial products, (2) the presence of ligands that might co‐purify with the CRP (as from biological fluids), and (3) damage to the molecule such as dissociation or aggregation, and (4) the presence of complement, all of which could cause this reagent to have varying biological effects. This system also employed human cells and allowed serum‐free conditions so that defined ligands for CRP could be tested. An accelerated conversion of monocytes to polarized macrophages has always been observed in this model, and that allowed the elimination of serum, with its constituent immunoglobulins (binding to Fc receptors), CRP‐binding moieties, and microparticles capable of converting pentameric to monomeric CRP.

Using contaminant free materials, we showed that CRP alone had no effect on M2 macrophage differentiation when used in a medium without serum. When combined with PC or phosphatidylcholine‐containing liposomes, that effect was highly promoted. Since CRP binds PC or other ligands on one face of the pentamer and FcγRs on the other face, it can bridge these molecules 11, 48, 49. These results indicate that either the avidity of interaction of CRP with FcγR or its affinity for individual FcγR may be increased by occupation of its opposite side with a PC‐containing ligand.

### Role of lipid oxidation

An important finding was that, while unoxidized phosphatidylcholine had no effect in this system, liposomes formed of the unoxidized form (POPC) with varying amounts of an oxidized form (PAzPC) depressed the background level of M2 macrophages, which was at least countered by CRP. Additionally, under conditions of molar equivalence with PAzPC, CRP addition led to the same increase in M2 macrophages as it did with PC. CRP only binds to the charged head group (PC) of a phospholipid when the lipid is oxidized 12, which explains its lack of an effect with the unoxidized POPC.

### Transition from an M1 to an M2 response can be incongruent with the T cell response

We characterized the cells undergoing monocyte differentiation to an M2 phenotype in this system and reproduced our previous findings that an early M1 response was followed by a later M2 response 50. That the M2 response in the present case was anti‐inflammatory was confirmed by the increased production of IL‐1RA. That it was mediated by the profibrotic cytokine IL‐13 (as previously noted by us and others 4, 51) was shown by mRNA levels and blocking experiments. We identified CD11b^+^ macrophages as one source for the IL‐13, introducing the possibility of an autocrine response.

Although the contribution of IL‐13 is usually associated with a Th2 response, we were surprised to find that, although we found increased levels of the Th2 transcription factor *GATA3* in response to PC plus CRP, there was no such response to liposomes plus CRP. Further, there was no increase in the expected number of CD4^+^ Th2 cells (CD294 and IL‐1R4 positive) in the cultures with liposome treatment and CRP. Concurrently, there was a vigorous Th1 response (*TBET* mRNA), especially in the cultures given liposomes, and that was not opposed by CRP.

An anti‐inflammatory T cell response from T cell subsets other than Th2 cells was ruled out by measuring aspects of Treg presence and products (FOXP3, IL‐10). IL‐1R1^+^ Treg cells were highly represented among CD4^+^ T cells in liposome‐treated cultures, but their numbers were not changed by CRP (see Supplementary Fig. S4c).

### FcγR signaling specificity is dependent on the species of CRP ligand

These results indicate that CRP induces anti‐inflammatory M2 differentiation despite a chronic Th1 response and relative failure of a Th2 response to liposomes containing oxidized lipids. We sought to explain the mechanism of M2 differentiation in the face of a proinflammatory T cell response, and investigated the role of CRP signaling via FcγR. We used blocking antibodies against individual FcγR and discovered that the CRP effect with PC was conveyed through FcγRI, whereas the response to CRP with liposomes was mediated through FcγRII. That these were both activating events was confirmed by the phosphorylation of SYK, an event downstream of activating FcγR (FcγRI and FcγRIIa) occupation 43. The differential activation of FcγRI or FcγRII by CRP with PC versus liposomes may be analogous to the higher affinity/avidity binding of monomeric immunoglobulin by FcγRI and immune complexes by FcγRII. In the case of CRP, its binding of the small molecule PC would not lead to complex formation, whereas its binding to the liposome at multiple sites could increase its avidity for FcγRII.

These data indicate that the binding of CRP to ligands and subsequent activation of FcγR may be solely responsible for the monocyte differentiation seen in this model. It has been reported that CRP binding to the fully oxidized lipid lysophosphatidylcholine impairs its affinity for FcγR, but maximum binding capacity was unchanged in that study, and subsequent signaling was not determined 52. In our study, we elected to use a less‐oxidized phosphatidylcholine (PAzPC) to avoid the detergent effects of lysophosphatidylcholine (and possible conversion of the pentameric to monomeric CRP) and to better approximate the degree of oxidation that may occur in vivo.

### Disparate macrophage and T cell responses

**Figure 9:**
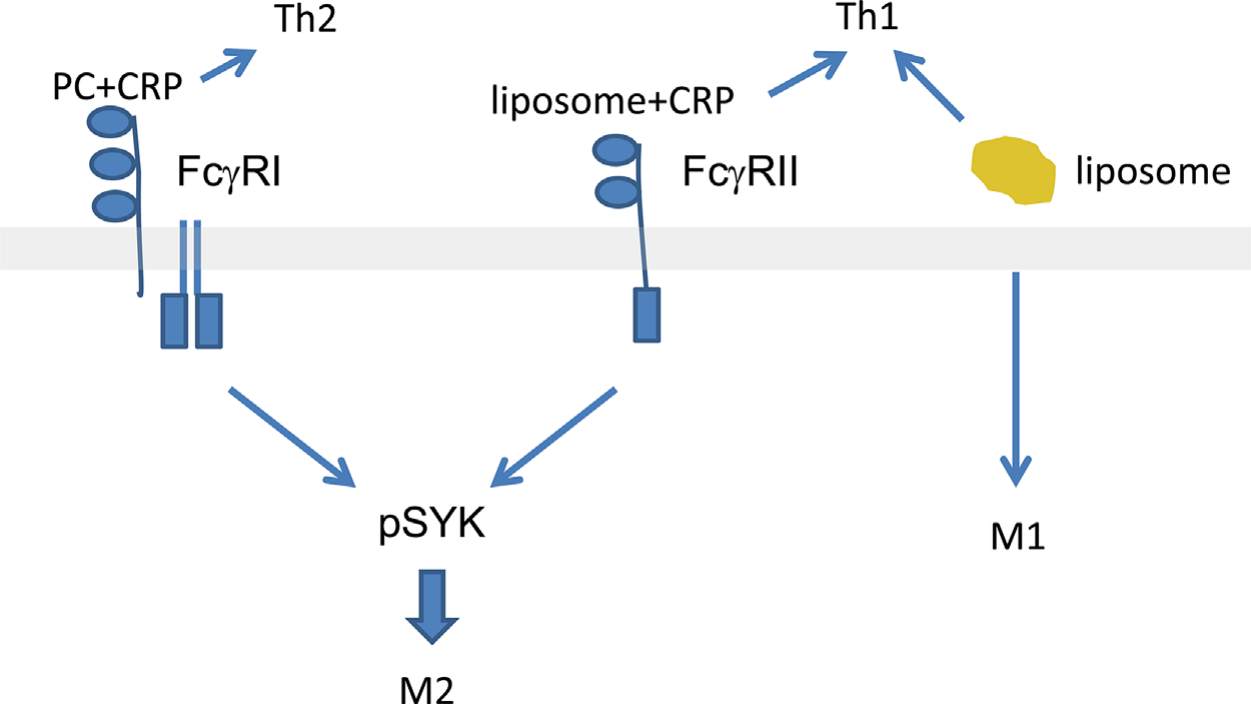
Schematic of results from this study, showing the binding of CRP and PC activating FcγRI leading to SYK phosphorylation and a switch from an M1 to an M2 macrophage phenotype, while liposome (lipid) complexed with CRP signaling leads to the same outcome via FcγRII. Liposomes, alone or bound to CRP, lead to a Th1 response.

We have already reported the correspondence of our model to our studies of cardiac fibrosis in the mouse 1, 2, 3. Here, we refined the TEM assay to permit the investigation of precise molecular configurations and cell subset analyses. It has been reported that oxidized lipids promote a proinflammatory Th1 and M1 response 53, 54. What is surprising is that CRP could shift the monocyte response to an M2 phenotype without affecting that T cell response. M2 macrophages have been observed to direct T cell activation and maturation 55, and so we expected a change from a Th1 to a Th2 response in cultures with CRP, especially since CRP has been reported to suppress Th1 cell differentiation 56. Others have shown that oxidized lipids induce maturation of naïve T cells to Th1 cells 57; thus it is possible that the Th1 response could become chronic or predominant based on continuous recruitment of new cells to a proinflammatory phenotype (Fig. 9). Given the independence of T cell and macrophage responses noted here, however, inflammation and fibrosis could coexist, as has been noted in atheromas 58, 59.

CRP may be relevant to both infection and sterile inflammation in atherosclerosis. Viable bacteria, including members of the oral, skin, and gastrointestinal flora, have been cultured from atheromas 60. Genomic analysis indicates the possibility of bacterial translocation between periodontal pockets and coronary arteries 61, 62 and there are differences in microbiota between symptomatic and asymptomatic plaques 63. Monocytes that engulfed bacteria are postulated to be the source of the translocation 61, and CRP may play a role in that process for PC‐bearing bacterial species. Our results here open the possibility that bacterial engulfment, depending on the receptors involved, may not result in a uniform or chronic proinflammatory response, but one that may develop into an anti‐inflammatory profibrotic phenotype.

### Potential pertinence to atherosclerotic cardiovascular disease

Macrophages take up oxidized lipids by a variety of receptors, including via CRP if the lipids bear PC. The resultant formation of foam cells may include their conversion into a pro‐fibrotic phenotype 64 as we have shown here. In that way and others, macrophages may be important effectors of the pathologic stages in atherosclerosis. While CRP is a commonly used biomarker for cardiovascular disease, its mechanistic link to the control of stability or lability in atherosclerotic plaques is not well defined. We do not presume to solve this issue. However, it should be noted that the CRP‐mediated effects described herein do not require T cell direction and result from interaction of ligand‐bound CRP with FcγR. The supply of these ligands is compatible with a chronic environmental exposure yielding an innate immune response. The resultant fibrosis in tissues would theoretically be an evolutionarily derived protective strategy.

Overall, these data from a simplified system indicate that, while CRP unbound to ligands may not have a significant effect on leukocyte biology, the type of ligand bound to it may be an important or even crucial factor in the clearance of complexes and the subsequent immune response. We have presented data from two different CRP ligands, phosphocholine and an oxidized phosphatidylcholine lipid, with different outcomes from each. PC and oxidized lipids arise from different origins, both of which have been linked to atherosclerosis via infections and/or lipid disorders. Although CRP‐related events occurring in vivo may be much more complex, this model presents a way to dissect molecular and cellular events that lead to fibrosis, a common component of many human disease states.

## Conflict of Interest

This research was conducted in the absence of any commercial or financial relationships that could be construed as a potential conflict of interest.

## Supporting information

Additional supporting information may be found in the online version of this article at the publisher's web‐site.

**Figure S1**. Microscopy and measurement of liposomes.
**Figure S2**. Counts of M2 macrophages with agents blocking IL‐13.
**Figure S3**. Immunofluorescence of cells treated with liposomes and CRP.
**Figure S4**. Measurement of other T cell subsets and products.
**Figure S5**. Flow cytometry histograms of T cell markers.
**Figure S6**. Immunofluorescence by flow cytometry of the CRP binding site on FcγRII.
