# Supplementary figures and images for: Phosphocholine‐containing ligands direct CRP induction of M2 macrophage polarization independent of T cell polarization: Implication for chronic inflammatory states
Source: Immun Inflamm Dis. 2016 Jun 20;4(3):274–88. doi: 10.1002/iid3.112 (PMC5004283; doi:10.1002/iid3.112)

Figure S1

(a) \_\_\_\_\_ (b)

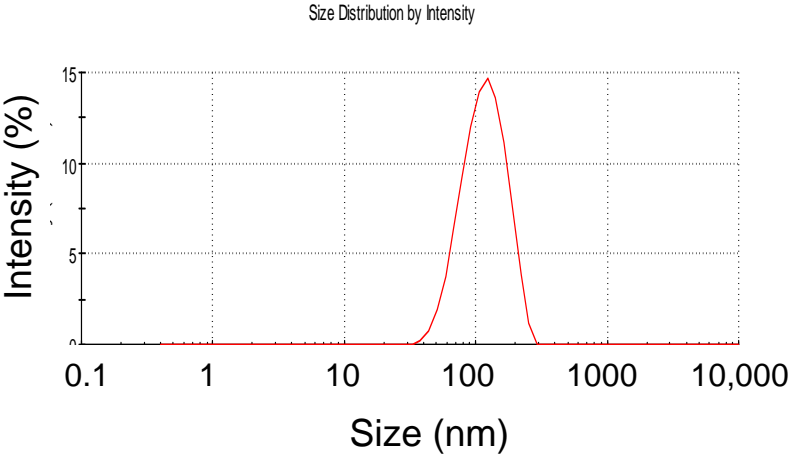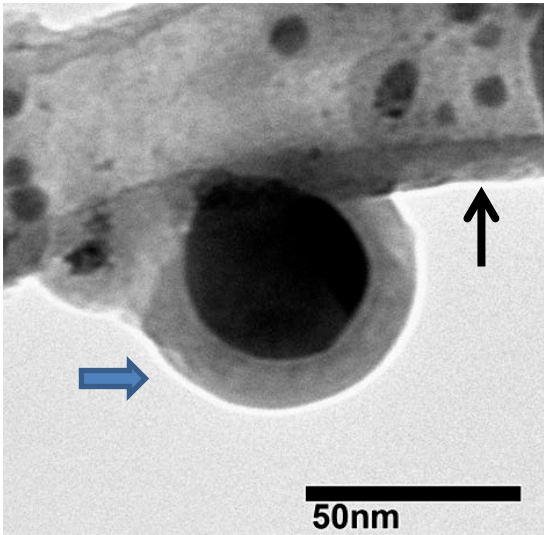

(c) \_\_\_\_\_ (d)

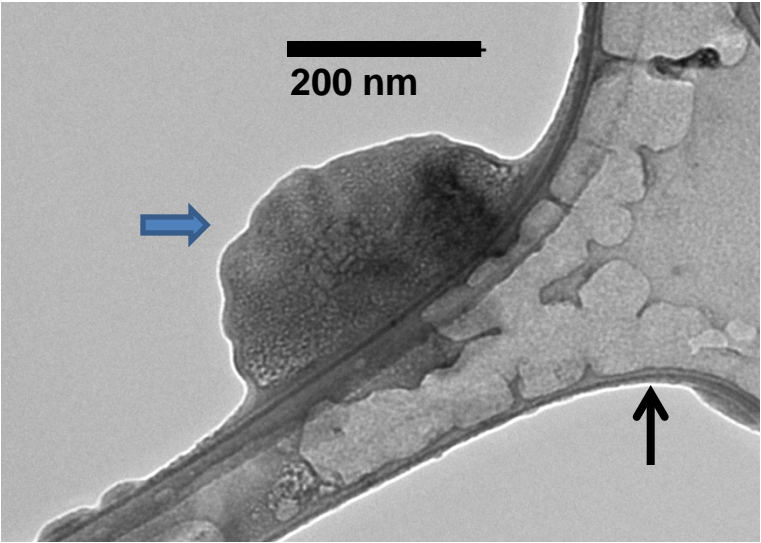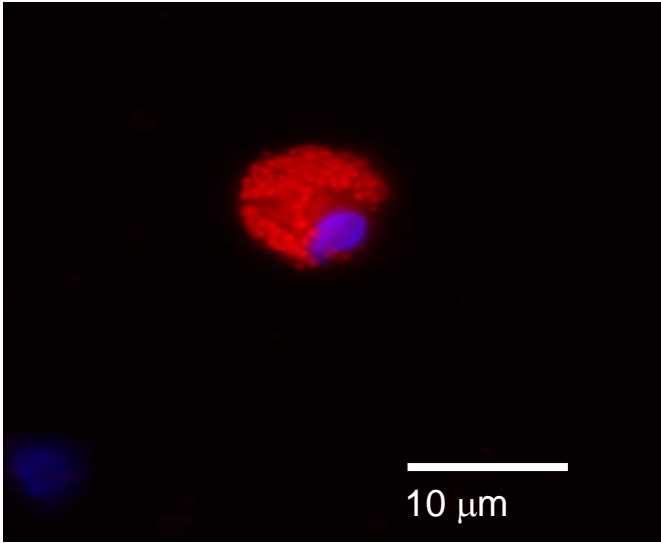

Figure S2

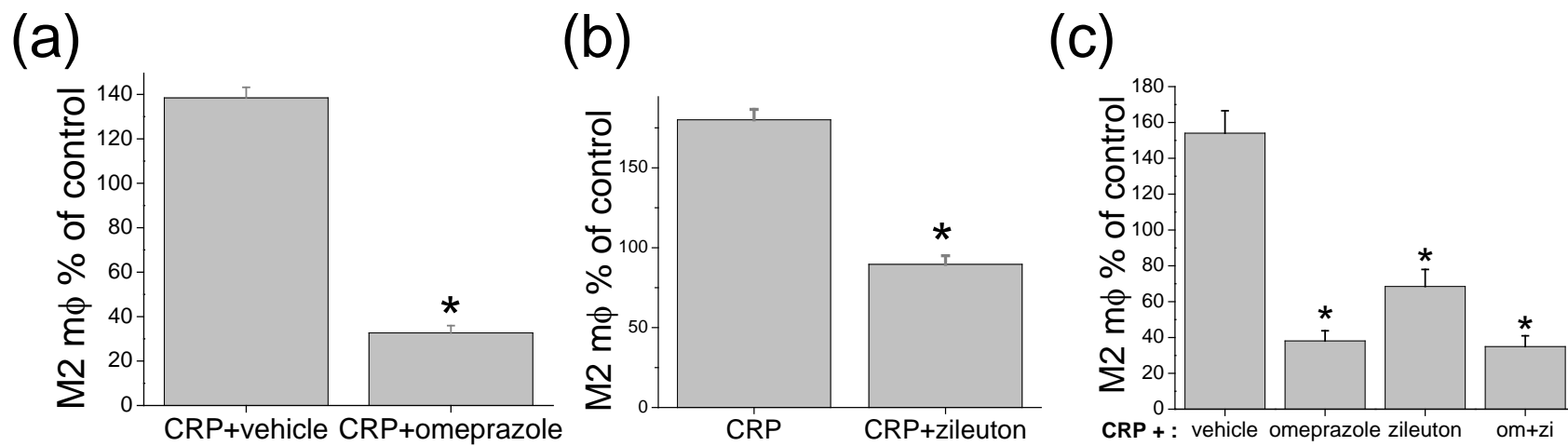

Figure S3

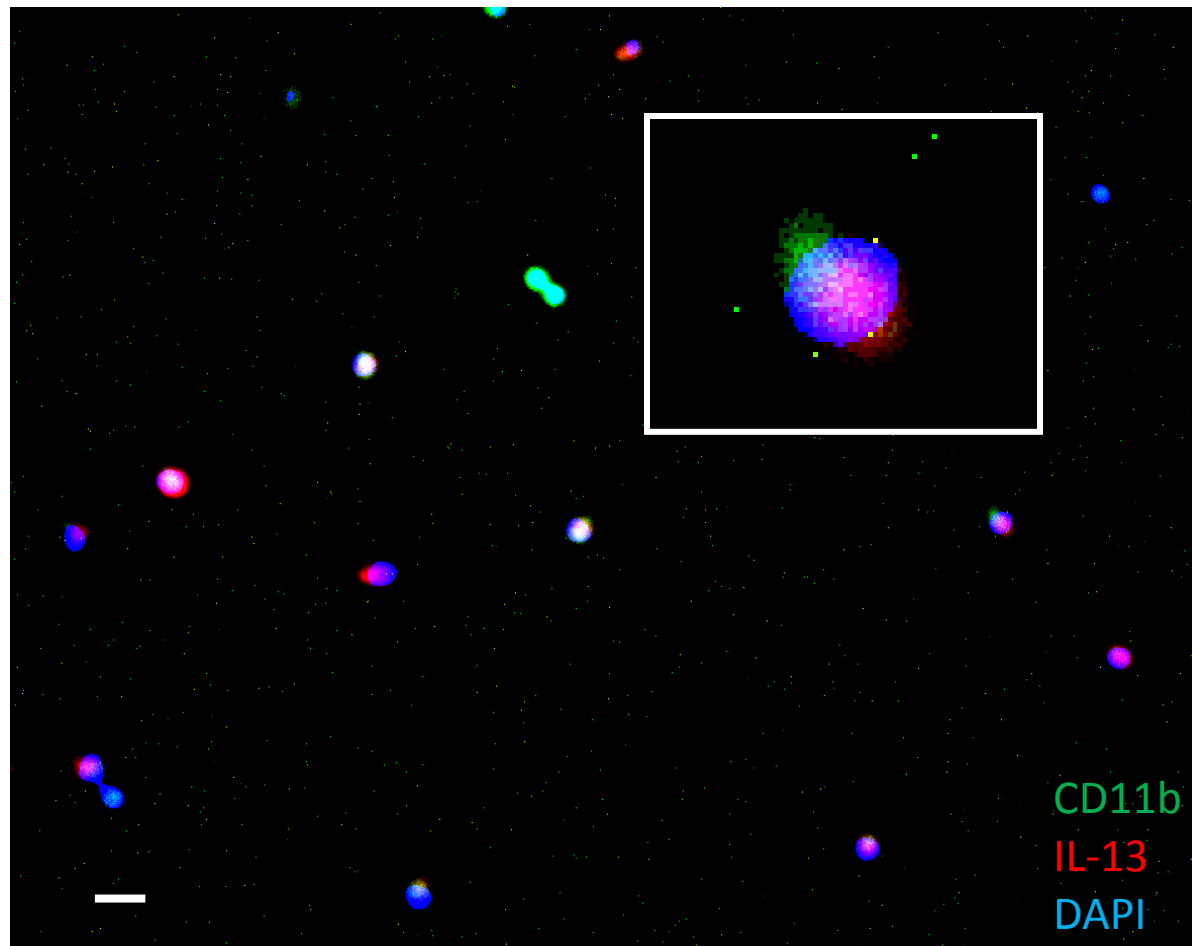

Figure S4

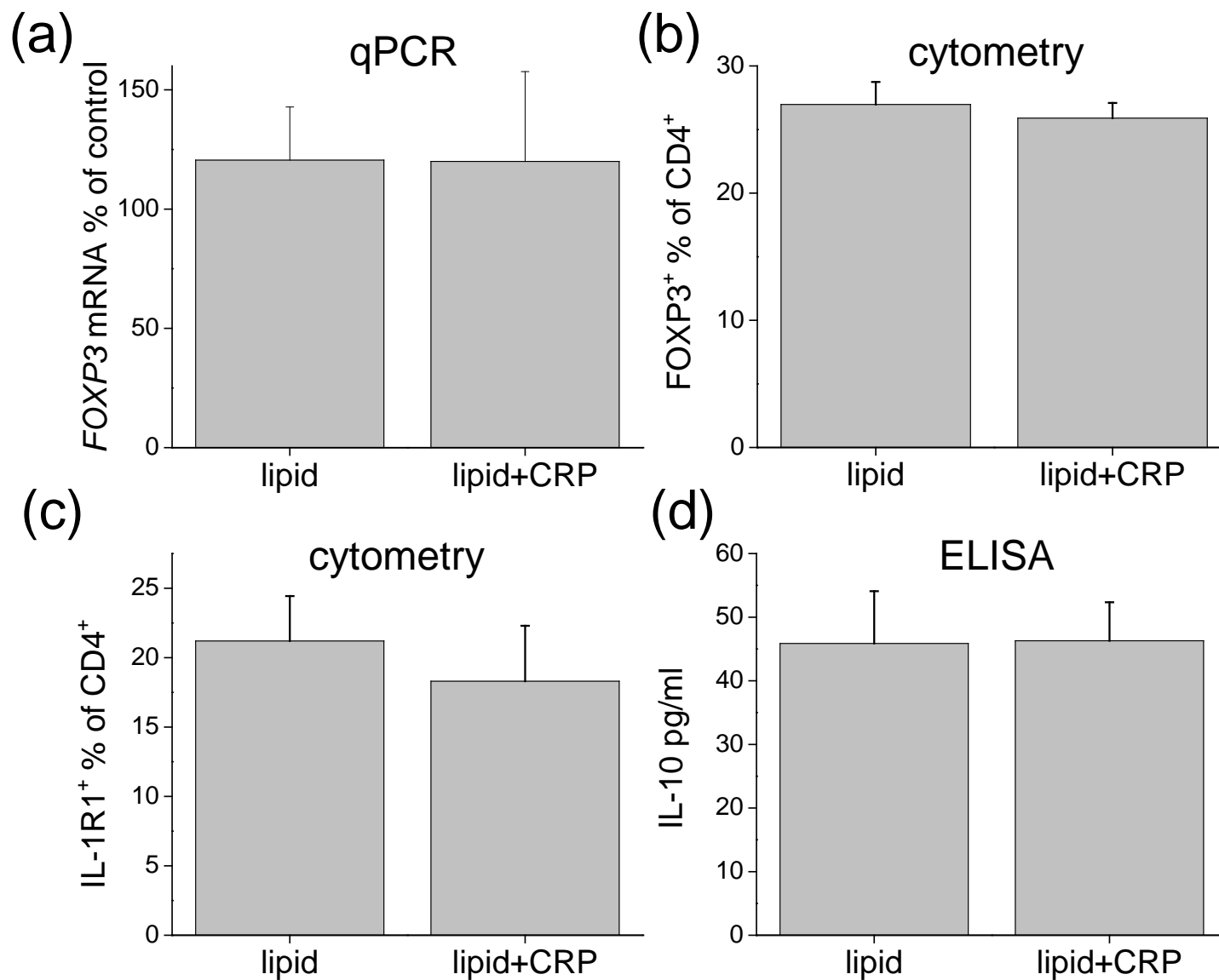

Figure S5

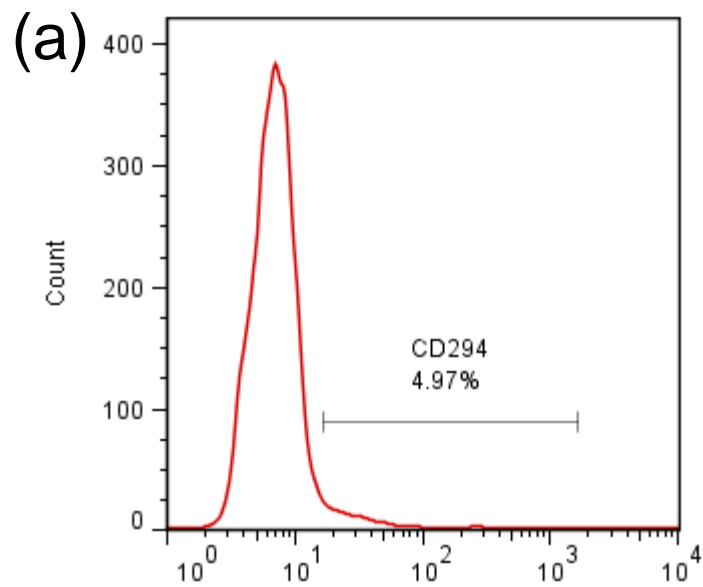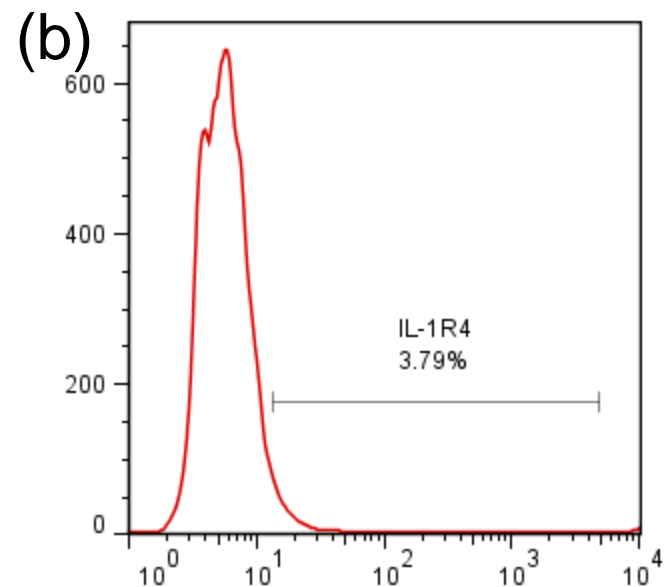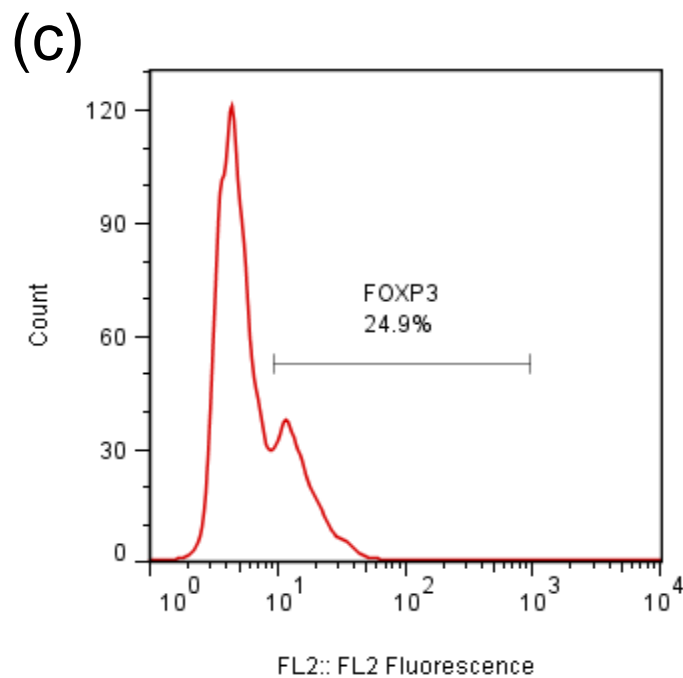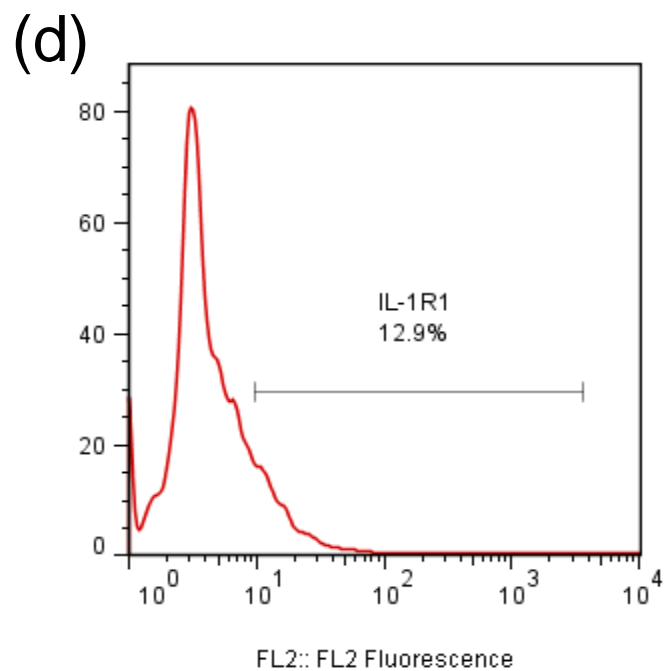

Figure S6

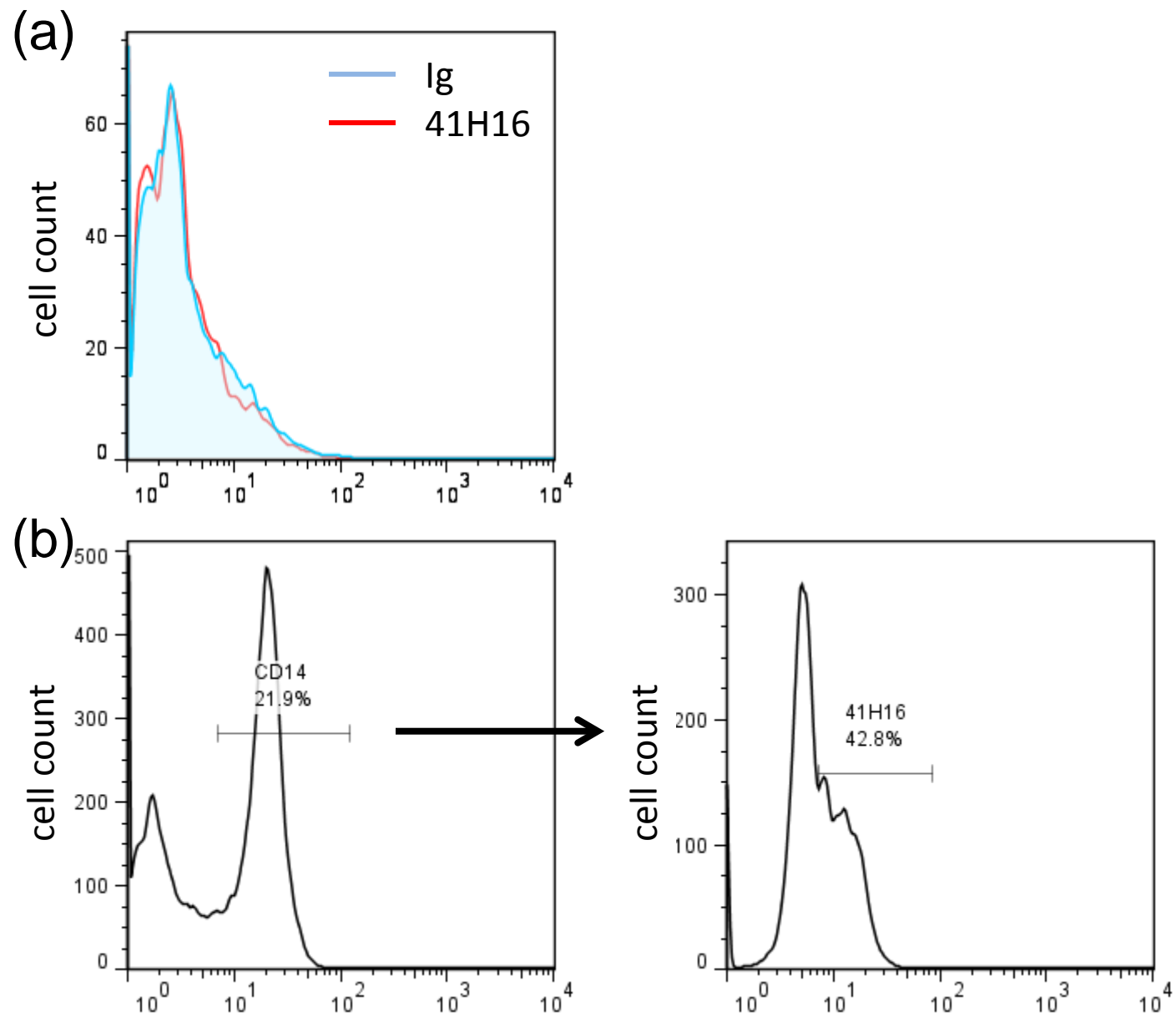

Supplement: Supplementary file 1 — Figure S1. Microscopy and measurement of liposomes. Figure S2. Counts of M2 macrophages with agents blocking IL‐13. Figure S3. Immunofluorescence of cells treated with liposomes and CRP. Figure S4. Measurement of other T cell subsets and products. Figure S5. Flow cytometry histograms of T cell markers. Figure S6. Immunofluorescence by flow cytometry of the CRP binding site on FcγRII. [file IID3-4-274-s001.pdf]
